# Accelerating Oncology Drug Reimbursement in Canada: Impact of the CDA-AMC Time-Limited Recommendation and pCPA Temporary Access Process

**DOI:** 10.3390/curroncol32040235

**Published:** 2025-04-17

**Authors:** Catherine Y. Lau, Arif Mitha, Allison Wills

**Affiliations:** 1 Independent Researcher, 158 Front Street E, Toronto, ON M5A 0K9, Canada; 2 20Sense Corp., Toronto, ON M4J 1G2, Canada; amitha@20sense.ca (A.M.); awills@20sense.ca (A.W.)

**Keywords:** drugs, cancers, patients, access, health Canada, notice of compliance with conditions (NOC/c), Canada’s drug agency (CDA-AMC), pan-Canadian pharmaceutical alliance (pCPA), time-limited reimbursement recommendation (TLR), pCPA temporary access process (pTAP)

## Abstract

The complex pathway for new drug reimbursement in Canada has been well documented. Drugs with promising early efficacy data may receive a Notice of Compliance with Conditions (NOC/c) from Health Canada. For oncology drugs that receive NOC/c, the pathway through positive review by Canada’s Drug Agency (CDA-AMC) and subsequent public reimbursement can take over 500 days. To address this challenge, in September 2023, CDA-AMC announced a new Time-Limited Recommendation (TLR) category, and in parallel, the pan-Canadian Pharmaceutical Alliance (pCPA) developed a set of principles and conditions for a Temporary Access Process (pTAP). This accelerated access pathway, the first of its kind in Canada, enables patients with advanced diseases to gain timely access to promising therapies while managing the uncertainties and risks associated with early approvals. This report provides a first assessment of the impact of the TLR-pTAP process on the reimbursement timelines for oncology drugs approved with NOC/c. **Methods**: The time from NOC/c approvals for oncology drugs between 1 January 2023 to 31 December 2024, to first provincial listings, and the timelines of the Health Canada, CDA-AMC, and pCPA review processes, were collected and evaluated. **Results**: Nine oncology NOC/c were granted during the selected period, of which three products, Columvi, Akeega, and Epkinly, received provincial listings, and the median time from regulatory approvals to provincial listings is 509 days (IQ range 306–544 days). One drug, Epkinly, has elected to adopt the TLR-pTAP pathway. Compared to the conventional reimbursement pathway—including for the drug Columvi, whose therapeutic profile is similar to that of Epkinly—the new pathway reduced the time to first provincial listing by over 200 days. A stepwise analysis indicates that the most significant accelerator within the TLR-pTAP pathway is the pCPA’s prioritization and processing of the file in parallel to the CDA-AMC’s health technology assessment (HTA) review process, rather than subsequently. Electing to file the HTA submission pre-NOC could have further accelerated timelines. No acceleration in each agency’s review time was observed. **Conclusions**: Participation in the TLR-pTAP pathway can help mitigate concerns over uncertainties associated with novel therapies while providing timelier access for patients with life-threatening diseases.

## 1. Introduction

To provide Canadian patients with severe and life-threatening diseases access to promising medications not yet available in Canada, Health Canada issued the first Notice of Compliance with Conditions (NOC/c) policy in 1998 and revised the document several times, with a final revision in 2009 [1]. This policy allows drug manufacturers to seek approval based on promising clinical evidence before the completion of confirmatory studies. To ensure the agreed-on conditions are met, the Qualifying Notice (QN) accompanying a NOC/c approval includes a letter of undertaking (LoU) documenting manufacturers’ responsibilities to obtain and report the confirmatory efficacy and safety evidence required for full approvals. Use of the NOC/c pathway is increasing—particularly for oncology drugs, having climbed from 11% in 1998–2001 to 94% in 2018–2021 [2]; a more recent publication confirms a similar increase from 2019 to 2024 [3].

**Figure 1:**
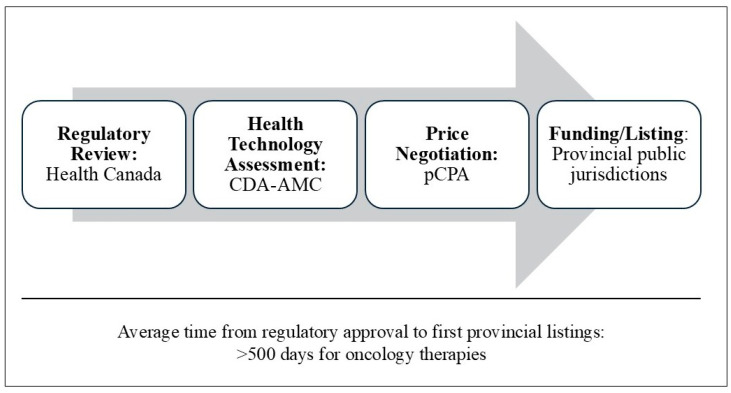
Steps in Canada’s public drug access process.

Because of uncertainties associated with conditional approvals, Canada’s Drug Agency (CDA-AMC) was initially reluctant to grant positive reimbursement recommendations [4]. From 2023 onwards, based on published evidence indicating that most oncology drugs receiving conditional or early approvals demonstrated positive data in confirmatory trials, CDA-AMC has increased the granting of positive recommendations to oncology drugs receiving NOC/c [2,3]. The next step of the public drug access process (Figure 1) is the pricing negotiation between manufacturers and the pan-Canadian Pharmaceutical Alliance (pCPA) culminating in a letter of intent (LoI), followed by the implementation of LoIs by provincial drug plans and resultant drug listing and funding [5]. For oncology drugs, the total time from regulatory approval through positive CDA-AMC review to provincial listings is over 500 days [6], and this lengthy timeline continues to impede critically ill cancer patients from receiving appropriate treatments [3].

Balancing early access for patients with clinical uncertainties and risks has become one of the most challenging tasks for health technology assessment and public funding agencies [4]. In September 2023, after much consultation earlier in the year, CDA-AMC announced a new Time-Limited Recommendation (TLR) category aiming to provide earlier access to promising new treatments approved via the NOC/c pathway [7,8]. In parallel, the pCPA developed a set of principles and conditions for a Temporary Access Process (pTAP) to inform the negotiation process of drug products that receive a positive CDA-AMC TLR [9]. Together, these processes form the first Canadian accelerated access pathway—a significant development in addressing timely patient access [10].

As outlined in the September 2023 guidance for issuing TLR, the new drug or indication approved must be authorized via the NOC/c pathway, have an evidence generation plan with phase 3 confirmatory trial(s) specified in the Health Canada QN to fill the data gap, and a commitment from the manufacturer to file a reassessment application within 270 calendar days after completion of the phase 3 trial (which must occur less than 3 years from the target expert committee meeting date) [8]. The pTAP process is mandatory for any product going through the TLR process and requires cost-effective pricing in any risk-shared agreement established with manufacturers [9]. The pCPA’s current practices with standard files occur subsequent to the HTA process, often with a significant time lag [11]. The pTAP principles and conditions outline the potential for the pCPA’s work to be conducted in parallel with CDA-AMC’s, noting that once the “initial economic report from the CDA-AMC” is available and “if the pCPA decides to pursue a negotiated agreement, a structured negotiation process will be initiated, prior to the TLR from the CDA-AMC” [9]. To evaluate the effectiveness of TLR, the guidance document indicates that the process will be re-evaluated after the first 3–5 recommendations or 18 months after the guidance issuance (which would be March 2025), whichever comes sooner [8]. pTAP is characterized as a pilot project, and as such will be subject to regular monitoring and assessment [9].

By the end of 2024, 15 months after the TLR guidance issuance, one new drug approved with NOC/c, Epkinly (epcoritamab), had entered the process. At the time of writing, a second drug had been announced as entering the process: a supplemental NOC/c approved on 17 January 2025, for Enhertu (trastuzumab deruxtecan) [12,13,14].

This report provides a comparison of the timeline of individual drug access process steps for Epkinly (epcoritamab), the first and only drug to have gone through TLR and pTAP, with similar steps for other NOC/c approved medicines that underwent the regular public drug access processes. The identified TLR and pTAP improvements could help to accelerate patients’ access to oncology drugs in Canada.

## 2. Methods

**Data Collection:**

Regulatory timeline: Health Canada Submission Under Review (SUR) was searched for antineoplastic drugs approved with NOC/c designations for new drugs and supplemental submissions between 1 January 2023 and 31 December 2024 [15]. For each NOC/c drug, the Summary Basis of Decision (SBD) database was searched for dates of submission, review acceptance, QN issuance, and approval [16]. Manufacturers were contacted for specific Health Canada dates when information was unavailable on the websites.

Health technology assessment timeline: CDA-AMC review report sites for the same products were searched, and review acceptance dates, draft review report, draft recommendation to the manufacturer, and final recommendation were recorded [17].

Price negotiation timeline: The pCPA website was searched for negotiation status, issuance dates for engagement letters, and the date of conclusion of negotiations with a LoI [18]. The pCPA was contacted for clarification on some dates. The cut-off date for collecting data from the CDA-AMC and pCPA websites was 31 January 2025.

Provincial funding timeline: Provincial listing status was obtained by searching the formulary websites of 13 Canadian public payer jurisdictions, including 10 provinces, one territory, the Non-Insured Health Benefits, and the Canadian Armed Forces Drug Benefit List. Drug plans were contacted by phone or email when information was unavailable on the websites. The first listing date for each applicable drug was captured for analysis [19,20,21].

**Data Analysis:** Each step of the drug approval process was assessed for duration, both individually and in relation to the target timelines of each agency (Table 1 and Supplemental Table S1).

Regulatory review time: The number of days in review by Health Canada was calculated as the time from file acceptance to QN issuance.

Health technology review time: The number of days in review by CDA-AMC was calculated from file acceptance to draft recommendation issued to the manufacturer.

Price negotiation review time: pCPA review times were calculated as follows (Supplemental Table S2): for phase 1 (initiation) and phase 2 (consideration), from the CDA-AMC final recommendation date to the issuance of the engagement, close, or hold letter to the manufacturer; for the TLR-pTAP product, as the pCPA engagement letter was issued prior to the final CDA-AMC recommendation, the calculation was from the issuance of the draft CDA-AMC review report [22]; phase 3 (negotiation) and phase 4 (completion), from the issuance of the engagement letter to the manufacturer to the issuance of the LoI or close letter to the manufacturer.

**Figure 2:**
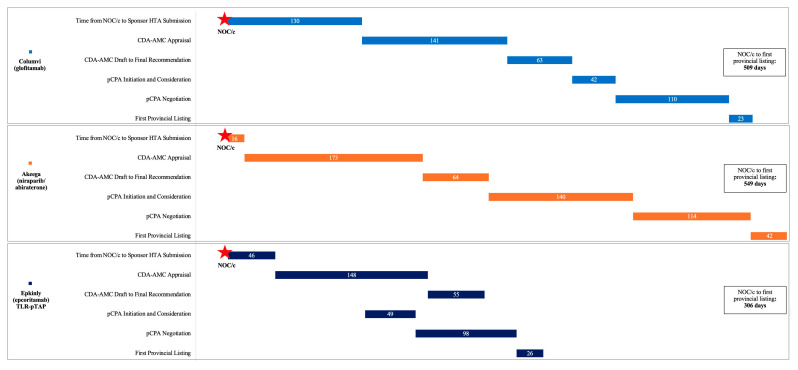
Aligned View of Time from NOC/c to First Provincial Listing for Columvi (glofitamab), Akeega (niraparib/abiraterone) and Epkinly (epcoritamab) (TLR-pTAP), in calendar days.

Product review time comparisons: for the three products that received provincial listings—Columvi, Akeega, and Epkinly—an assessment was conducted of the aligned view of time from NOC/c to first provincial listing, which allowed the identification of gaps or overlaps between processes (Figure 2). The time between NOC/c issuance and CDA-AMC dates for file acceptance, and the time between CDA-AMC final recommendation date and the date of pCPA publication of the issuance of the engagement letter, were used to calculate additional time gaps or overlaps between individual agency processes.

Days in review calculation: An Excel formula (fx = DATADIF) was used to calculate the days in review and between submissions. All analyses were performed descriptively for the report.

## 3. Results

Table 1 lists all oncology drugs approved with NOC/c from 1 January 2023 to 31 December 2024. This period was selected for review as Health Canada, CDA-AMC, and pCPA started to engage stakeholders in accelerated reimbursement pathway discussions around the beginning of 2023, with a draft document released in March 2023 and final guidance released in September 2023 [7,8].

Between 1 January 2023 and 31 December 2024, nine oncology drug submissions received NOC/c approvals, which included seven new drugs, one combination of two marketed oncology drugs, and one supplemental: Carvykti (ciltacabtagene autoleucel) [23,24,25], Columvi (glofitamab) [19,26,27,28], Akeega (niraparib, abiraterone acetate combination) [20,29,30,31], Tecvayli (teclistamab) [32,33,34], Epkinly (epcoritamab) [19,21,35,36,37], Elrexfio (elranatamab) [38,39,40], Talvey (talquetamab) [41,42], Imdelltra (tarlatamab) [43,44,45], and Lynparza (olaparib, supplemental) [46]. As of 31 January 2025, only three products—Columvi, Akeega, and Epkinly—received provincial listings. The remaining drugs either did not submit to CDA-AMC (Lynparza), are in active negotiation with pCPA (Carvykti, Tecvayli, Elrexfio, Imdelltra), or received a ‘do not reimburse’ recommendation from CDA-AMC (Talvey) (Table 1, Supplemental Table S1). One product, Epkinly, entered the TLR-pTAP pathway.

For the three products that received provincial listings (Columvi, Akeega, and Epkinly), Figure 2 shows the timelines from NOC/c approvals to the first provincial listings. For Columvi and Akeega, which went through the standard pathway, the times from NOC/c to the first provincial listings were 509 and 549 days, respectively. This aligns with the typical timeline for oncology drugs of 581 days [6]. Epkinly, which went through the TLR-pTAP pathway, exhibited the shortest time from NOC/c to the first provincial listing at 306 days—a significant (275-day) acceleration from the norm for oncology drugs.

As observed in the following results, the duration of each of the individual drug access processes at Health Canada, CDA-AMC, and pCPA was unchanged from regular processing times, and did not have a significant impact on the accelerated timelines for Epkinly, the TLR-pTAP product.

For all nine products, Health Canada review times were mainly on target (200 days) [47], except for Akeega, a new combination of two existing oncology products, and Lynparza, a supplemental NOC/c, which had long review times of 297 and 307 days, respectively. The reasons for the long review time are unknown; however, for both products, only regulatory decision summaries are available online. Therefore, any delays due to company-requested pauses would not be available. For Carvykti, a complex CAR-T cell therapy, the review time was 287 days; however, three regulatory pauses were requested by the manufacturer; therefore, the actual Health Canada review time was on target at 199 days [48]. The review time for Epkinly, the TLR-pTAP product, was 200 days.

The next step in the drug review process is health technology assessment (HTA) by CDA-AMC, which accepts files for review once submitted by the manufacturer. For the drugs considered in this analysis, this ranged from −125 days prior to regulatory approval to 130 days after regulatory approval (Table 1 and Supplemental Table S1). Two products, Carvykti [23,24,25] and Elrexfio [38,39,40], used pre-NOC filings (parallel reviews between Health Canada and CDA-AMC) (Table 1), as evidenced by the −125 and −12 days between NOC/c approval and submission to CDA-AMC, respectively. Imdelltra was accepted in −14 days despite being designated as “post NOC” at filing [45]. Time gaps between regulatory approval and acceptance of the CDA-AMC submission were observed for all three products that went on to receive provincial listings: for Epkinly, the TLR-pTAP product, the delay was 46 days, for Columvi, 130 days, and for Akeega, 16 days. Despite Epkinly not electing to follow a pre-NOC pathway, which could have further accelerated timelines, 46 days is shorter than the time gap for Columvi, at 130 days, and partially contributed to Epkinly’s faster listing (Figure 2).

CDA-AMC review times for all products fell within the pre-set targets of ≤180 days [49]. Columvi’s review time was the shortest at 141 days, and Akeega’s was the longest at 173 days. The review time for Epkinly, the TLR-pTAP product, was mid-range at 148 days. Time from CDA-AMC drafts to final recommendations ranged from 55 to 64 days (55 days for Epkinly).

The pCPA’s target time to initiation and consideration is ≤40 business days (about 55 calendar days) from CDA-AMC’s final recommendation date [50]. Among the six products with data for this time interval, the median was 126 (from 42 days for Columvi to 177 days for Carvykti), and Epkinly was 49 days. The fact that the pCPA initiated the Epkinly file much earlier than other submissions—116 days prior to the CDA-AMC final recommendation—and conducted their work in parallel with the CDA-AMC process significantly contributed to Epkinly’s faster listing (Figure 2).

For the three products that completed pCPA negotiations (Columvi, Akeega, and Epkinly), the negotiation time ranges were 110, 114, and 98 days, respectively, all below the pCPA’s target of ≤90 business days (about 125 calendar days) [50]. The negotiation for Epkinly, the TLR-pTAP product, was the shortest at 98 days (Table 1).

The time from the end of pCPA negotiation to first provincial listings was similar for the three drugs: 23 days for Columvi (Quebec), 42 days for Akeega (Quebec), and 26 days for Epkinly (Quebec and Ontario [19,20,21]), the TLR-pTAP product, which had limited impact on the total timeline for approval.

## 4. Discussion

**Delays in cancer patients accessing new, life-saving medications in Canada:**

Over the past decade, new drug discoveries in oncology have driven innovation in the field of medicine. This development, accompanied by a progressive regulatory approval strategy led by the US FDA and followed by many developed countries such as the European Union, the United Kingdom, Canada, and Australia, has dramatically increased the treatment armamentarium for cancer patients, especially for those with life-threatening disease. The traditional reimbursement pathway is struggling to keep pace with the influx of new therapies, resulting in patients lacking access to appropriate and affordable treatments.

Among the Organisation for Economic Co-operation and Development (OECD) HTA countries surveyed in a recent review by Cowling et al., all have some form of early reimbursement pathway, allowing oncology patients to access public funding for new oncology medications, except Germany and Canada [51]. The delays have led to Canadian cancer patients being deprived of effective treatments.

**Improvements Achieved Through the TLR and pTAP Processes:**

To address this gap, CDA-AMC and pCPA announced the implementation of a time-limited recommendation and temporary reimbursement pathway (TLR and pTAP) in September 2023 [8,9].

As reported in the Results, upon stepwise review of the TLR-pTAP process, review times by all three agencies (Health Canada, CDA-AMC, and pCPA) approximated those for other NOC/c products and fell within preset targets, and none of the review agencies compromised their review times (Table 1). What explains Epkinly’s accelerated timeline to its first listing is that the pCPA initiation and consideration phase happened in parallel to the CDA-AMC review. The question remains whether the pCPA will continue to prioritize TLR-pTAP files ahead of other files in the queue, as appeared to be the case with Epkinly, and if a more explicit prioritization policy will be added to the pTAP principles.

Parallel reviews between regulatory and HTA agencies are not new. For drugs submitted to Health Canada and CDA-AMC, approximately half leverage the pre-NOC parallel review process [52]. Epkinly did not elect to follow the pre-NOC parallel review process, which could have further accelerated its timelines, and, as such, the TLR review at CDA-AMC was initiated after NOC/c. Nevertheless, the 46-day gap between NOC/c and CDA-AMC acceptance is one of the shorter gaps observed and contributed to Epkinly’s faster listing (Figure 2). Manufacturers have the opportunity to consider leveraging the pre-NOC parallel review process when preparing TLR-pTAP files. Notably, the CDA-AMC is now seeking to enable further use of the pre-NOC parallel process with its Target Zero and rolling review improvement initiatives [52].

The timeline comparisons for two of the three products that successfully received funding, Epkinly and Columvi, as described in Figure 2, further demonstrate the impact of the temporary process. With both molecules being bispecific monoclonal antibodies targeting CD3 and CD20 antigens, they share similar mechanisms of action and therapeutic similarities in terms of risks, uncertainties, and proximity in the submission schedule. Stimulating CD3 antigens on T cells enables them to attach to the B cell antigen CD20, leading to the death of cancerous B cells [53,54]. Both therapies are indicated for Diffuse Large B Cell Lymphoma (DLBCL), and both were approved based on phase I/II data. Epkinly is formulated as a subcutaneous injection, whereas Columvi requires infusion. Despite Epkinly being filed to Health Canada 6 months after Columvi (Supplemental Table S1), these two drugs received their first provincial listings at the same time. Thus, entering into the TLR-pTAP process could potentially reduce approximately 6 months or 30% of the total review time.

**Implications of the Research Findings and Recommendations:**

Fifteen months after the publication of TLR-pTAP, only one drug has gone through the process. More recently, the oncology drug Enhertu has been submitted for the TLR-pTAP pathway for a new indication [13,14]. The supplemental drug submission was accepted for review by Health Canada on 14 May 2024 and received NOC/c on 17 January 2025; CDA-AMC accepted the submission on October 1, 2024, as part of a pre-NOC review. pCPA announced it received a letter of intent (which starts the initiation and consideration phase) on 17 January 2025 [14]. This is the same day as the NOC/c issuance and preceded both the draft and final CDA-AMC recommendations, representing a notable acceleration of the pCPA process. If pCPA follows its target of ≤140 days for the entire process, the time from NOC/c to the first provincial listing could be less than 200 days, a significant improvement from current statistics. If this prioritization of pTAP files at the pCPA can be applied to future reimbursement reviews, the TLR-pTAP pathway will have successfully achieved its goals of significantly accelerating patient access, albeit for a limited range of drugs for now.

As illustrated by this analysis, there is an opportunity to expand this expedited access process to a more significant number of oncology products. Approaches to consider include expanding the current eligibility of the TLR and pTAP processes to allow for more drug files; incorporating greater flexibility into the Phase III clinical trial requirements by allowing the use of Phase III data where the patient population, line of therapy, and/or indication do not fully align with the Phase II trial data provided in the original HTA submission; and accepting real-world evidence (RWE) as a supplement to clinical trial data [10]. Furthermore, the pCPA could consider leveraging the pTAP process for non-TLR files—for example, files that receive NOC through Health Canada’s priority review pathway, which is reserved for products “intended for the treatment, prevention or diagnosis of serious, life-threatening or severely debilitating illnesses or conditions with a high unmet need…where there is no existing drug on the Canadian market with the same profile or where the new product represents a significant improvement in the benefit/risk profile over existing products” [55]. In 2024, of the 115 new and supplemental drug submissions in Canada, 20 leveraged the priority review pathway [15]. This must be balanced with consideration of the impact of processing more files through accelerated access pathways: could the pCPA support a higher volume of such priority files, and how would this impact other files?

While the first TLR-pTAP file has completed the drug access component of the process, the full TLR-pTAP process will only be completed following confirmatory data from the manufacturer and a subsequent HTA to permanently establish or discontinue funding. Evaluation of the pathway’s success will be possible only at that time. Disclosure of CDA-AMC’s and pCPA’s evaluation processes and metrics would increase transparency and understanding of the impact of this pathway. Questions remain: Is the current rate of one TLR-pTAP file per year a success? How many patients have ultimately benefited from this process, and is that sufficient?

## 5. Conclusions

While assessing the pathway’s proficiency falls outside the scope of this report, its initial uptake by only one product is disappointing. The findings from this research—that an accelerated access pathway in Canada can indeed expedite patient access when all parties agree to work together—are encouraging. An assessment of the first Canadian accelerated access pathway, CDA-AMC’s TLR and pCPA’s pTAP processes, suggests that prioritization of appropriate files and collaboration among reimbursement and funding agencies would shorten the time for oncology patients to access new therapies. Further process improvements are encouraged, including for drugs for rare and other difficult-to-treat diseases.

## Figures and Tables

**Table 1:** Time from NOC/c to First Provincial Listing and Review Times by Health Canada, CDA-AMC, and pCPA as of 31 December 2024, in calendar days.

Drugs (Generic Name) Approved with NOC/c From 1 January 2023 to 31 December 2024	Time From NOC/c Issuance to First Provincial Listings	Health Canada Time from File Accepted for Review to NOC/c QN Issuance	Time from NOC/c Issuance to CDA-AMC Acceptance for Review	Time from CDA-AMC Acceptance for Review to Draft Recommendation	Time from CDA-AMC Draft to Final Recommendation	Time for pCPA Initiation and Consideration	Time for pCPA Negotiation	Time from End of pCPA Negotiation to First Provincial Listing
**Columvi** **(glofitamab)**	Completed 509	199	130	141	63	42	110	23
**Akeega** **(niraparib/abiraterone)**	Completed 549	297	16	173	64	140	114	42
**Epkinly** **(epcoritamab)** **TLR-pTAP**	Completed 306	200	46	148	55	49 ^a^	98	26
**Carvykti** **(ciltacabtagene autoleucel)**	Active >722	287	−125	165	57	177	Active	NA
**Tecvayli** **(teclistamab)**	Active >555	200	70	147	56	167	Active	NA
**Elrexfio** **(elranatamab)**	Active >422	199	−12	151	56	112	Active	NA
**Imdelltra** **(tarlatamab)**	Active >142	198	−14	146	NA	NA	Active	NA
**Talvey** **(talquetamab)**	NA	NA ^b^	15	160	NA (CDA-AMC DNR, no submission to pCPA)			
**Lynparza** **(olaparib)** **supplemental**	NA	307	NA (No submission to CDA-AMC or pCPA)					

NA = Not Available, QN = Qualifying Notice, DNR = Do Not Recommend, ^a^ = Calculated from the Draft CDA-AMC review report to the issuance of the pCPA engagement letter, ^b^ = QN issuance date not available.

## Data Availability

The original contributions presented in the study are included in the article; further inquiries can be directed to the corresponding author.

## Supplementary Materials

The following supporting information can be downloaded at: https://www.mdpi.com/article/10.3390/curroncol32040235/s1, Table S1: Key submission filing, acceptance and decision dates recorded by Health Canada, CDA-AMC, pCPA and first provincial listing dates as of 31 January 2025, for oncology drugs approved with NOC/c between 1 January 2023 to 31 December 2024. Table S2: Target timelines for each step of the drug approval process by agency for Health Canada, Canada’s Drug Agency (CDA-AMC) and the pan-Canadian Pharmaceutical Alliance (pCPA).

## Abbreviations

The following abbreviations are used in this manuscript:

NOC/c	Notice of Compliance with Conditions
CDA-AMC	Canada’s Drug Agency
TLR	Time-Limited Recommendation
pCPA	pan-Canadian Pharmaceutical Alliance
pTAP	pan-Canadian Pharmaceutical Alliance Temporary Access Process
QN	Qualifying Notice
LoU	Letter of undertaking
LoI	Letter of intent
SUR	Submission Under Review
SBD	Summary Basis of Decision
HTA	Health technology assessment
OECD	Organisation for Economic Co-operation and Development
DLBCL	Diffuse Large B Cell Lymphoma
