# Supplementary material for: Accelerating Oncology Drug Reimbursement in Canada: Impact of the CDA-AMC Time-Limited Recommendation and pCPA Temporary Access Process
Source: Curr Oncol. 2025 Apr 17;32(4):235. doi: 10.3390/curroncol32040235 (PMC12025630; doi:10.3390/curroncol32040235)
Supplement: Supplementary file 1 [file curroncol-32-00235-s001.zip › SUPPLEMENTAL TABLE S1.pdf]

<u>Supplemental Table S1</u>: Key submission filing, acceptance and decision dates recorded by Health Canada, CDA-AMC, pCPA and first provincial listing dates as of January 31, 2025, for oncology drugs approved with NOC/c between Jan. 1, 2023, to Dec. 31, 2024.

	**Health Canada**			**CDA-AMC**			**pCPA**		**Provincial Listing**
	**Submission accepted**	**NOC/c Qualifying Notice issued**	**NOC/c date**	**CDA-AMC submission accepted date**	**Draft recommendation issues to sponsor**	**Final recommendation posted**	**Engagement letter issued**	**Negotiation process concluded**	**First provincial listing date (province)**
Carvykti (ciltacabtagene autoleucel)	1-Apr-22	13-Jan-23	9-Feb-23	7-Oct-22	21-Mar 23	17-May-23	10-Nov-23	Active	NA
Columvi (glofitamab)	25-Jul-22	9-Feb-23	24-Mar-23	1-Aug-23	20-Dec-23	21-Feb-24	3-Apr-24	22-Jul-24	14-Aug-24 (QC)
Akeega (niraparib/ abiraterone)	5-Aug-22	29-May-23	12-Jun-23	28-Jun-23	18-Dec-23	20-Feb-24	9-Jul-24	31-Oct-24	12-Dec-24 (QC)
Tecvayli (teclistamab)	1-Dec-22	19-Jun-23	26-Jul-23	4-Oct-23	28-Feb-24	24-Apr-24	8-Oct-24	Active	NA
Epkinly (epcoritamab)*	10-Feb-23	29-Aug-23	13-Oct-23	28-Nov-23	24-Apr-24	18-Jun-24	12-Apr-24	19-Jul-24	14-Aug-24 (ON, QC)
Elrexfio (elranatamab)	14-Apr-23	30-Oct-23	6-Dec-23	24-Nov-23	23-Apr-24	18-Jun-24	8-Oct-24	Active	NA
Talvey (talquetamab)	13-Sep-23	NA	30-Apr-24	15-May-24	22-Oct-24	DNR	NA	NA	NA
Imdelltra (tarlatamab)	10-Jan-24	26-Jul-24	11-Sep-24	28-Aug-24	21-Jan-25	NA	NA	NA	NA
Lynparza (olaparib)**	10-Aug-22	13-Jun-23	11-Jul-23	No Submission	NA	NA	NA	NA	NA

* Epkinly's *Draft Canada's Drug Agency review report(s) provided to sponsor for comment* date is 23-Feb-24; **Supplemental NOC/c; NA=Not Available; DNR=Do Not Reimburse recommendation by CDA-AMC
