# Supplementary material for: Accelerating Oncology Drug Reimbursement in Canada: Impact of the CDA-AMC Time-Limited Recommendation and pCPA Temporary Access Process
Source: Curr Oncol. 2025 Apr 17;32(4):235. doi: 10.3390/curroncol32040235 (PMC12025630; doi:10.3390/curroncol32040235)
Supplement: Supplementary file 1 [file curroncol-32-00235-s001.zip › SUPPLEMENTAL TABLE S2.pdf]

<u>Supplemental Table S2</u>: Target timelines for each step of the drug approval process by agency for Health Canada, Canada's Drug Agency (CDA-AMC) and the pan-Canadian Pharmaceutical Alliance (pCPA)

Agency	Target Timelines for Review	Start	End
**Health Canada**[1]	**200 calendar days** for the NOC/c review process	Upon acceptance at screening	Upon issuance of the NOC/c Qualifying Notice
**CDA-AMC**[2]	**≤180 calendar days**	The date the file is accepted for review	The date the draft recommendation is issued to the sponsor and drug programs
**pCPA**[3-5*]	Phase 1. Initiation, and<br>Phase 2. Consideration:<br>**≤40 business days; approx. 55 calendar days**	<u>Standard files</u>:<br>From the final HTA recommendation date.<br><u>pTAP files:</u><br>From when the CDA-AMC initial economic report is issued.	Issuance of the engagement, close or hold letter to the manufacturer
	Phase 3. Negotiation, and<br>Phase 4. Completion:<br>**≤90 business days; approx. 125 calendar days**	Issuance of the engagement letter to the manufacturer	Issuance of LOI or close letter to the manufacturer

** Health Canada and CDA-AMC report their targets in calendar days. pCPA reports their targets in business days. In this table, pCPA business days have been converted to calendar days.*

Sources:
1. https://www.canada.ca/content/dam/hc-sc/migration/hc-sc/dhp-mps/alt_formats/pdf/prodpharma/applic-demande/guide-ld/compli-conform/noccg_accd-eng.pdf, accessed Nov. 8. 2024.
2. https://www.cdaamc.ca/sites/default/files/Drug_Review_Process/Drug_Reimbursement_Review_Procedures.pdf, accessed Nov. 8. 2024.
3. https://www.pcpacanada.ca/sites/default/files/eng/pCPA_Brand_Process_Guidelines.pdf, accessed Nov. 8. 2024.
4. Email to the pCPA, Question about October 2024 dashboard, January 23, 2025.
5. pCPA Temporary Access Process (pTAP). https://www.pcpacanada.ca/ptap, March 2, 2025.
